# Antibodies against phosphorylcholine are not altered in plasma of patients with Alzheimer’s disease

**DOI:** 10.1186/s12883-015-0260-1

**Published:** 2015-02-05

**Authors:** Edina Silajdžić, Maria Björkqvist, Oskar Hansson

**Affiliations:** Brain Disease Biomarker Unit, Department of Experimental Medical Science, Wallenberg Neuroscience Center, Lund University, Lund, Sweden; Clinical Memory Research Unit, Department of Clinical Sciences Malmö, Lund University, Malmö, Sweden; Memory Clinic, Skåne University Hospital, Malmö, Sweden

**Keywords:** Anti-phosphorylcholine, Alzheimer’s disease, Dementia, Biomarker

## Abstract

**Background:**

Phosphorylcholine is one of the major epitopes of oxidised low density lipoprotein. Low levels of IgM antibodies against phosphorylcholine (anti-PC) are associated with development of myocardial infarction and stroke. It has been shown that patients with Alzheimer’s disease and other dementias have significantly lower serum anti-PC levels compared to controls, suggesting that low levels of atheroprotective anti-PC may play a role in AD and dementia.

**Methods:**

We quantified levels of anti-PC levels using an ELISA in plasma from 176 controls, 125 patients with Alzheimer’s disease, 19 patients with vascular dementia and 63 patients with other dementias.

**Results:**

We observed similar plasma anti-PC levels in controls, patients with Alzheimer’s disease, and other dementias.

**Conclusions:**

Our data suggests that anti-PC is not useful as a biomarker for Alzheimer’s disease.

## Background

Accumulation of oxidised low density lipoprotein (OxLDL) is a hallmark of atherosclerosis, the underlying cause of cardiovascular disease (CVD). Phosphorylcholine (PC) is a hapten-like epitope that is exposed on OxLDL, some microorganisms and apoptotic cells [1,2]. PC may play an important role in the atherogenic and proinflammatory effects of OxLDL [3]. It has been shown that IgM antibodies against phosphorylcholine (anti-PC) have anti-inflammatory properties and that low levels of anti-PC predict the development of stroke and myocardial infarction [3].

CVD and Alzheimer’s disease (AD) may be related through common underlying factors such as oxidative stress and inflammation [4,5]. As a result, there has recently been an increased interest in whether vascular pathology contributes to AD [6-14]. Several vascular risk factors, such as hypertension, atherosclerosis and hypercholesterolemia have been reported to be associated with development of AD [6-14]. Moreover, it has been shown that patients with AD have reduced serum levels of atheroprotective anti-PC compared to controls and that the likelihood of having dementia or AD was doubled for individuals with anti-PC values in the lowest quartile, suggesting that low levels of anti-PC may play a role in AD and dementia [15]. To determine whether the anti-PC levels in plasma are reduced in individuals with AD and dementia, we quantified plasma levels of anti-PC in a cohort comprising of 176 controls, 125 patients with AD and 82 patients with other dementias.

## Methods

### Collection and processing of human plasma samples

Plasma samples were obtained from patients at the Memory clinic, Skåne University Hospital, Malmö, Sweden. All patients underwent thorough standard examinations conducted by a trained physician, including neurological, physical and psychiatric examinations. All patients diagnosed with AD had to meet the DSM-IIIR criteria of dementia [16] and the criteria of probable AD defined by NINCDS-ADRDA [17]. Patients diagnosed with vascular dementia (VaD) fulfilled the DSM-IIIR criteria of dementia and the requirements for probable VaD by NINDS-AIREN [18] or the recommendations by Erkinjuntti et al. for VaD of the subcortical type [19]. For the diagnosis of dementia with Lewy Bodies or frontotemporal dementia, the consensus criteria by McKeith et al. [20] and McKhann et al. were used [21], respectively. The healthy volunteers had no memory complaints or other cognitive symptoms, and no active neurological diseases. Non-fasting plasma was collected between 9 and 11 am. After venipuncture, blood was collected in tubes prepared with EDTA to prevent coagulation. Samples were centrifuged and plasma was removed from the tubes leaving 1 ml of plasma to avoid contamination of plasma with blood cells. Within one hour of venipuncture the plasma was frozen in polypropylene tubes at – 80°C until biochemical analysis. The study was conducted in accordance with the Helsinki Declaration and the study procedure was approved by the ethics committee of Lund University, Sweden. All controls gave written informed consent. The patients underwent plasma sampling as part of the clinical routine investigation and in conjunction with this procedure they gave oral informed consent for future use of their banked plasma samples for research. This was documented in the patients’ medical records. Moreover, all individuals were later on instructed to retract their permission, if they had changed their minds, as instructed in local press advertisements. The procedure for use of plasma samples obtained in clinical routine after oral consent was approved by the ethical committee (reference number “Dnr 289/2008”).

### Analysis of plasma anti-PC

Anti-PC levels were quantified in plasma diluted 1:101 in accordance with the manufacturer’s recommendations using CVDefine (Athera Biotechnologies, Stockholm, Sweden), an indirect, noncompetitive, enzyme immunoassay for quantitative determination of anti-phosphorylcholine IgM antibodies in human serum or plasma. The assay is based on PC antigen covalently linked to bovine serum albumin coated onto 96-well microtiter plates and PC-specific IgM antibodies present in the plasma sample bind to the antigen. The detection limit of CV Define is 0.5 U/ml and the inter-assay coefficient of variation is below 8%. This assay has previously been used in numerous publications in both serum and plasma [15,22-25].

### ApoE4 allele genotyping

APOE (gene map locus 19q13.2) genotyping was performed using TaqMan® Allelic Discrimination technology (Applied Biosystems, Foster City, CA). Genotypes were obtained for the two SNPs that are used to unambiguously define the ε2, ε3, and ε4 alleles (rs7412 and rs429358).

### Analysis of Aβ42, total tau, phosphorylated tau and albumin

The CSF samples were collected into polypropylene tubes at the memory clinics in Malmö according to routine clinical procedures, and the procedure and the analysis followed the Alzheimer’s Association Flow Chart for lumbar puncture [26]. After centrifugation, the CSF samples obtained were frozen at −80°C. The samples were analysed using commercially available enzyme-linked immunosorbent assays (ELISAs) (Innogenetics, Ghent, Belgium) to determine the levels of total tau, Aβ42 and tau phosphorylated at Thr181 (P-tau) (INNOTEST® hTAU Ag, β-AMYLOID (1–42) and PHOSPHO-TAU (181P), respectively). All analyses were performed by board-certified laboratory technicians using procedures accredited by the Swedish Board for Accreditation and Conformity Assessment (SWEDAC). CSF and plasma albumin levels were analysed on a Cobas C501 analyser (Roche Diagnostics, Mannheim, Germany).

### Statistical analysis

Inter-group differences in plasma anti-PC were identified using one-way analysis of variance (ANOVA). Linear regression analyses were used to investigate associations between plasma anti-PC and Mini-Mental-State Examination (MMSE), age and gender in all groups, whereas correlation between plasma anti-PC and cerebrospinal fluid (CSF)/serum albumin ratio was only performed in the AD group. Linear regression analyses between plasma anti-PC and CSF biomarkers Aβ42, total tau and phosphorylated tau were only performed in a subset of samples (36 control and 31 AD samples). In addition, we performed multivariable linear regression with plasma anti-PC as the dependent variable and CSF Aβ42, total tau, and phosphorylated tau concentrations or MMSE as independent variables, adjusting for presence of the ApoE4 allele, age, gender and body mass index (BMI). ApoE4 allele status and CSF biomarker data was available for 36 control and 31 AD samples.

## Results

**Figure 1:**
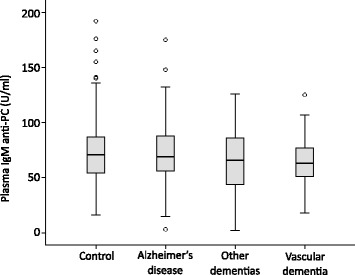
**Box-and-whisker plots of plasma anti-PC concentration.**

We measured plasma levels of anti-PC in subjects with AD, vascular dementia, “other” dementias (including 34 dementia with Lewy Bodies, 17 frontotemporal dementia, and 12 Parkinson’s disease with dementia subjects) and age-matched controls. In Table 1 we present the demographic data and plasma anti-PC levels. As shown in Figure 1, there was no significant difference in levels of anti-PC between controls and subjects with AD, vascular dementia or “other” dementias (ANOVA, p 0.23). The mean and SD of anti-PC levels in the “other” dementia groups was as follows: dementia with Lewy bodies 62 ± 25 U/ml, frontotemporal dementia 59 ± 28 U/ml and Parkinson’s disease with dementia 87 ± 37 U/ml.

**Table 1:** **Subject demographics and plasma anti-PC levels**

	**Controls**	**AD**	**Other dementias** ^**1**^	**VaD**
**n**	176	125	63	19
**Mean age, years (range)**	74 (62–99)	76 (56–87)	71 (45–85)	76 (56–87)
**% of females**	63	72	59	68
**MMSE [SD]**	29 [1.3]	21 [4.8]	21 [5.7]	22 [3.8]
**Mean anti-PC, U/ml [SD]**	73.3 [29.9]	73.4 [25.4]	66.2 [29.8]	65.3 [26.6]

^1^Other dementias: dementia with Lewy Bodies (n = 34), frontotemporal dementia (n = 17) and Parkinson’s disease with dementia subjects (n = 12).

We also examined a possible correlation between plasma anti-PC and MMSE, a measure of global cognition, in each individual group (control, AD, VaD and “other” dementias), however, there was no correlation between anti-PC levels and MMSE scores in any group tested. Also, there was no correlation between plasma anti-PC and age or gender. Additionally, we examined whether a correlation could be seen between plasma anti-PC and CSF biomarkers Aβ42, total tau and phosphorylated tau in a subset of control and AD samples (n = 36 and n = 31, respectively). The only significant association found was between CSF total tau and plasma anti-PC in the control group only: CSF total tau levels statistically significantly predicted plasma anti-PC [F(1, 34) = 7.114 p < 0.012, R^2^ = .173]. The association between plasma anti-PC and CSF total tau levels in the control group remained significant after controlling for age, gender, BMI and presence of the ApoE4 allele (p < 0.008). In the AD group, we also examined the correlation between plasma anti-PC and CSF/serum albumin ratio, which is a measure of blood brain barrier integrity, however, no correlation was found.

## Discussion

Antibodies to PC, an epitope on OxLDL, are present in normal healthy individuals. Previous studies have shown that anti-PC levels below 17 U/ml in middle-aged men and women predicted a statistically significant risk for CVD (myocardial infarction and stroke) independent of other risk factors, such as blood pressure, cholesterol and smoking [15,22-25]. A recent study investigated serum anti-PC levels in AD and dementia and reported that elderly individuals with AD and dementia have lower levels of natural IgM anti-PC antibodies than age and gender matched controls and decreasing anti-PC levels linearly increased the odds ratio of AD [15]. Since low IgM anti-PC is an independent risk marker for development of CVD [22-25], the authors hypothesised that the immune related alterations involved in CVD could also play a role in AD. In the current study, we also measured anti-PC levels in elderly subjects with AD and other dementias, however, we did not observe reduced levels of circulating anti-PC in patients with dementia, nor did we observe any association between anti-PC concentration and the extent of cognitive impairment as measured by MMSE. Thus, our results do not substantiate the observations of Eriksson et al. [15].

Since we used the same assay as was used in the Eriksson et al. study [15], the contrasting results are not due to differences in kit manufacture. Also, since anti-PC levels are not correlated with age or gender, the discrepancies in the results are not likely to be due to any differences in age or gender distribution. Moreover, the gender distributions in the control and AD cohorts are very similar in our study and in the Eriksson et al. paper [15]. One of the reasons why we could not replicate the findings of Eriksson et al. [15] may be because the cohorts used in the two studies are at different disease stages. The mean MMSE score in our AD and mixed dementia group was 21 ± 5, however, since this information is not available in the Eriksson et al. paper [15], it is difficult to directly compare the disease stage in these two studies. Our cohort is on average younger than that in the Eriksson et al. study [15] (76 ± 7 and 82 ± 5 years, respectively, for the AD group), thus, it may be possible that our cohort is at an earlier disease stage. The fasting status of the samples is sometimes the reason for a discrepancy in results, however, in the Eriksson et al. study the association between anti-PC and dementia was not found to be affected by the fasting status, thus, the fasting status is unlikely to be the reason why we could not replicate the findings of Eriksson et al. [15]. In the present study IgM anti-PC was quantified in plasma samples, whereas in the Eriksson et al. study [15], it was quantified in serum. The levels of some analytes vary significantly between serum and plasma. The higher levels of anti-PC in plasma in this study compared to those in serum in the Eriksson et al. study [15] suggest that this may be a case for anti-PC. However, a previous study found similar IgM anti-PC levels in serum and plasma and showed that repeated freeze-thawing had no effect on IgM anti-PC levels [27].

It is possible that anti-PC levels may be different in different clinical populations since differences may exist between laboratories and cohorts with respect to sample handling and storage, diet, use of medication, BMI, blood pressure and other unknown factors.

## Conclusions

Many studies have suggested a link between vascular pathology and AD [6-14] and thus it is likely that alterations involved in CVD do indeed play a role in dementia, however, the results of the current study suggest that plasma anti-PC might not play a pivotal role in the molecular mechanisms underlying AD.

## Abbreviations

AD: Alzheimer’s disease
ANOVA: Analysis of variance
Anti-PC: IgM antibodies against phosphorylcholine
BMI: Body mass index
CSF: Cerebrospinal fluid
CVD: Cardiovascular disease
ELISA: Enzyme-linked immunosorbent assay
MMSE: Mini-Mental-State Examination
OxLDL: Oxidised low density lipoprotein
VaD: Vascular dementia
